# C–H Bond Activation by the Excited Zinc Atom: Gas-Phase Formation of Methylzinc Hydride (HZnCH_3_) Based on Multireference Second-Order Perturbation Theory and Coupled Cluster Calculations

**DOI:** 10.1021/acsomega.1c04531

**Published:** 2021-09-06

**Authors:** Jerzy Moc

**Affiliations:** Faculty of Chemistry, Wroclaw University, F. Joliot-Curie 14, 50-383 Wroclaw, Poland

## Abstract

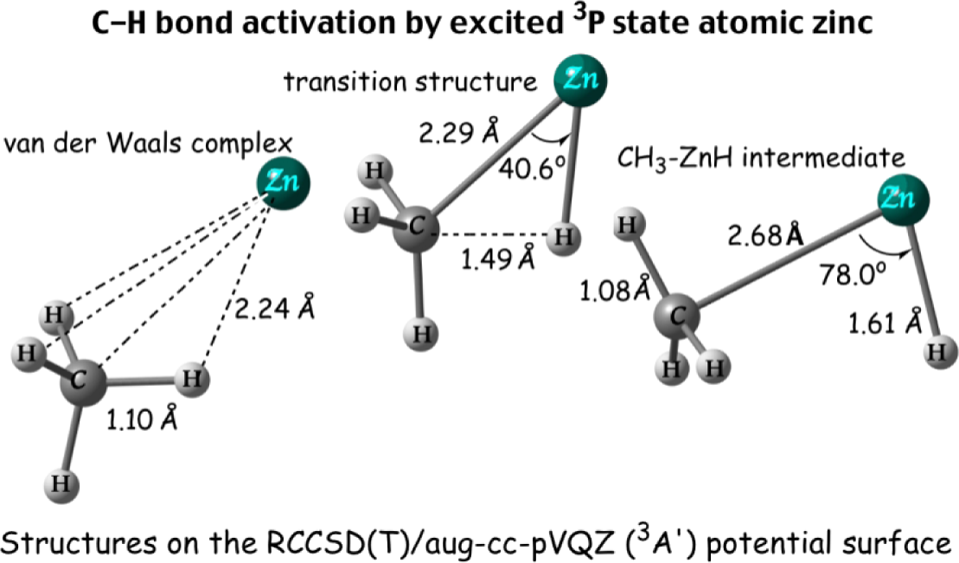

The pioneering spectroscopic observations of the methylzinc hydride [HZnCH_3_(X^1^A_1_)] molecule were reported previously by the Ziurys group [*J. Am. Chem. Soc.***2010**, 132, 17186–17192], and the possible formation mechanisms were suggested therein, including those with the participation of excited zinc atoms in reaction with methane. Herein, the ground singlet state and the lowest excited triplet state potential energy surfaces of the Zn + CH_4_ reaction have been explored using high-level electronic structure calculations with multireference second-order perturbation theory and coupled cluster singles and doubles with perturbative triples (CCSD(T)) methods in conjunction with all-electron basis sets (up to aug-cc-pV5Z) and scalar relativistic effects incorporated via the second-order Douglas–Kroll–Hess (DK) method. Based on the ab initio results, a plausible scenario for the formation of HZnCH_3_(X^1^A_1_) is proposed involving the activation of the C–H bond of methane by the lowest excited ^3^P state atomic zinc. Calculations also highlight the importance of an agostic-like Zn···H–C interactions in the pre-activation complex and good agreement between the structure of the HZnCH_3_(X^1^A_1_) molecule predicted at the DK-CCSD(T)/aug-cc-pVQZ-DK level of theory and that derived from rotational spectroscopy, as well as the discrepancies between the ab initio and density functional theory predictions.

## 1 Introduction

The activation of C–X (X = H, halogen) bonds in homogeneous systems is a research field relevant to important industrial processes.^1^ For X = H, the catalytic activation of C(sp^3^)–H bonds presents a much bigger challenge compared to that of C(sp^2^)–H bonds.^2,3^ In particular, splitting of the strong and nonreactive carbon–hydrogen bond in methane is thought to be the key step for methane conversion;^4^ in the present work, we use the “organometallic definition” of C–H bond activation as referring to “the formation of a carbon–metal bond by cleavage of a carbon–hydrogen bond”.^2,3^

In 2010, a methylzinc hydride molecule, HZnCH_3_, was synthesized in the gas phase in a DC discharge by the reaction of zinc vapor with methane in the presence of Ar gas and identified by using millimeter/submillimeter direct-absorption and Fourier-transform microwave spectroscopic techniques as described by Ziurys and co-workers.^5^ From the rotational constants (B) of the seven HZnCH_3_ isotopologues, an *r*_o_ structure of the HZnCH_3_ molecule in the ground X^1^A_1_ electronic state was derived.^5^ In addition to the gas-phase reaction of atomic zinc with methane,^5^ the Zn/CH_4_ system was the subject of the low-temperature matrix isolation infrared (IR)^6,7^ and vacuum ultraviolet spectroscopy^8^ investigations. These investigations employed irradiation to accomplish the activation of the C–H bond in methane by zinc atoms.^6−8^ On the theoretical side, the Zn + CH_4_ reaction was studied^9,10^ using mostly density functional theory (DFT) and with an ab initio multireference configuration interaction (MRCI) plus the multireference second-order Møller–Plesset perturbation (MR/MP2) (MRCI- MR/MP2) method employing effective core potentials^11^ (we will refer to the results of the previous theoretical studies of Zn + CH_4_ when appropriate).

The electric discharge-induced reaction of zinc vapor with methane studied by Ziurys and co-workers^5^ is of importance in organometallic synthesis and serves as a model system for C–H bond activation. Although the possible formation mechanisms of the methylzinc hydride (HZnCH_3_(X^1^A_1_)) have been discussed by Ziurys and co-workers^5^ and other workers,^10,11^ including that of insertion of the zinc atom in the electronically excited (^1^P or ^3^P) state into the carbon–hydrogen bond of methane, the detailed mechanism by which CH_4_ is activated by atomic zinc in the lowest excited ^3^P state along with the subsequent formation of HZnCH_3_(X^1^A_1_) has not been sufficiently addressed. To provide more insight into this issue, we have embarked on a theoretical study to investigate the ground singlet state and the lowest excited triplet state potential energy surfaces of the Zn + CH_4_ reaction by using high-level single-reference and multireference ab initio methods (detailed in Section 4). We have also examined the sensitivity of the energetics of the Zn + CH_4_ system to the scalar relativistic effects.

## 2 Results and Discussion

### 2.1 Ground (^1^S) State and Excited (^3^P and ^1^P) States of Atomic Zinc

To prove the accuracy of the methods used, the calculated relative energies of the ground ^1^S(3d^10^4s^2^) state and the excited ^3^P(3d^10^4s^1^4p^1^) and ^1^P(3d^10^4s^1^4p^1^) states of the Zn atom are compared with the experimental data^12,13^ in Tables 1 and S1. These tables show that the scalar relativistic effects, included using the second-order Douglas–Kroll–Hess (DK) Hamiltonian, cause the significant increase in the energy separation between the ^1^S and ^3^P states, thereby improving the accuracy of the estimate. For instance, the scalar relativistic effects are found to be 14.6, 18.4, and 18.0 kJ/mol with MCSCF(2,4)/aug-cc-pVTZ, MCQDPT2(2,4)/aug-cc-pVTZ, and CASPT2(2,4)/aug-cc-pVTZ, respectively, where MCSCF denotes multiconfigurational self-consistent field wave function, and MCQDPT2 and CASPT2 are two different implementations of the multireference second-order perturbation theory (MRPT2); the last three methods use the active space consisting of 2 electrons in 4 orbitals, (2,4), arising from Zn 4s4p orbitals. Our most accurate estimate of the relative energy for the ^1^S and ^3^P states of the Zn atom (Table 1), derived from the DK-(R)CCSD(T)/aug-cc-pV5Z-DK calculations of 389.9 kJ/mol [CCSD(T) signifies coupled-cluster theory with single, double, and perturbative triple excitations, with “R” indicating its spin-restricted variant used for the triplet], is consistent with earlier report^14^ and the experimental value of 391.2 kJ/mol.^12,13^Table 1 further shows that, at the DK-CASPT2(2,4)/aug-cc-pVTZ-DK level, the energy separation between the ^1^S and ^1^P states of 569.9 kJ/mol is somewhat overestimated compared to the experimental^12,13^ result of 559.4 kJ/mol. It is, however, the lowest excited ^3^P state of atomic zinc of primary importance from the point of view of the reaction mechanism considered in the present work.

**Table 1:** Relative Energies (kJ/mol) of the Ground ^1^S(3d^10^4s^2^) State and the Excited ^3^P(3d^10^4s^1^4p^1^) and ^1^P(3d^10^4s^1^4p^1^) States of the Zn Atom Calculated Using Single-Reference^a^ and Multireference Methods

method	^1^S(3d^10^4s^2^)	^3^P(3d^10^4s^1^4p^1^)	^1^P(3d^10^4s^1^4p^1^)
Non-Relativistic			
MCQDPT2(2,4)^b^/aug-cc-pVTZ	0.0	366.1	560.2
CASPT2(2,4)^b^/aug-cc-pVTZ	0.0	364.0	552.7
CCSD(T)/aug-cc-pV5Z	0.0	371.5	
With Scalar Relativistic Effects via Second-Order DK			
DK-MCQDPT2(2,4)^b^/aug-cc-pVTZ-DK	0.0	384.5	578.2
DK-CASPT2(2,4)^b^/aug-cc-pVTZ-DK	0.0	382.0	569.9
DK-CCSD(T)/aug-cc-pV5Z-DK	0.0	389.9	
exp.^c^	0.0	391.2^d^	559.4

a For Zn(^3^P), this implies RCCSD(T).

b Active space used in the MCQDPT2 and CASPT2 calculations was 2 electrons in 4 orbitals, (2,4), arising from Zn 4s4p orbitals.

c Reference (12).

d The spin–orbit average excitation energy (derived from Moore’s tables^12^).

Before proceeding with the Zn + CH_4_ system, we note that the remainder of this paper is organized as follows. First, we report on a pathway of the reaction of ground-state atomic zinc with methane; this includes a comparison of the equilibrium structure and vibrational frequencies of the insertion product with the available experimental data. After that, we describe a pathway for the reaction of excited ^3^P state atomic zinc with methane. A plausible mechanism for the formation of HZnCH_3_(X^1^A_1_) is next presented, followed by conclusions. All the relative energies quoted below are zero-point vibrational energy (ZPVE)-corrected, except for those given in Table 6.

### 2.2 van der Waals Complex Formation and Insertion Step for the Reaction of Ground-State Atomic Zinc with Methane

**Figure 1:**
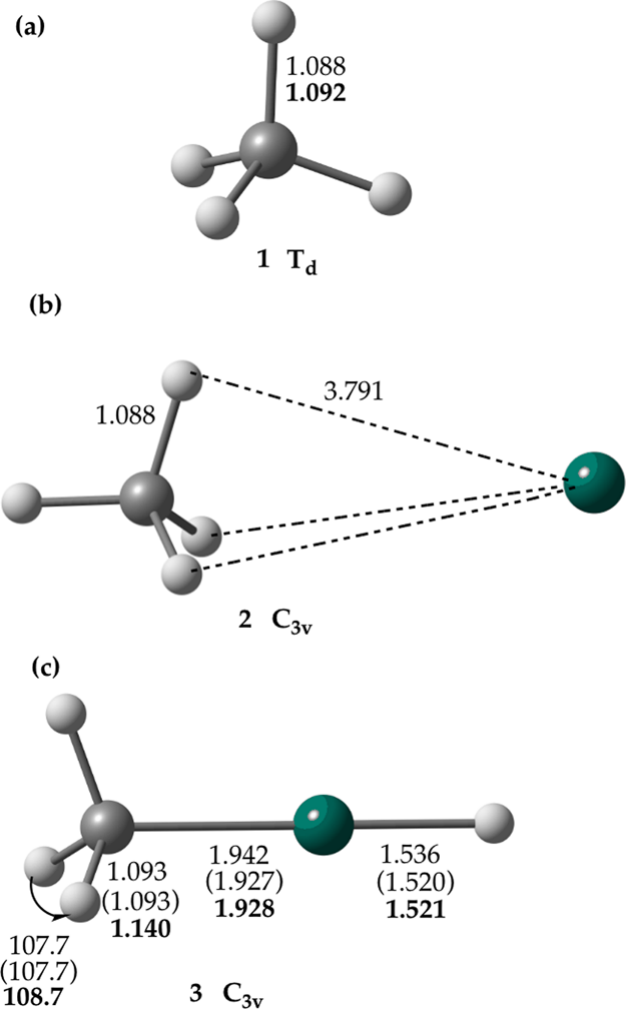
Structures of (a) reactant CH_4_ (**1**), (b) vdW complex Zn···η^3^-H_3_CH (**2**), and (c) insertion product HZnCH_3_ (X^1^A_1_) (**3**) of the reaction of ground-state atomic zinc with methane optimized at the CCSD(T)/aug-cc-pVQZ level. For **3**, structural parameters shown in parentheses are from the DK-CCSD(T)/aug-cc-pVQZ-DK geometry optimization. Bold font indicates the experimental bond distances from ref (27) (CH_4_**1**) and ref (5) (HZnCH_3_(X^1^A_1_) **3**). Distances are in Å and angles are in degrees.

The reaction of ground-state atomic zinc with methane **1** (Figure 1a) entails formation of a van der Waals (vdW) complex Zn···η^3^-H_3_CH **2** of *C*_3v_ symmetry (Figure 1b) whose binding energy (1.5 kJ/mol, Table 2) is found to be comparable to that of the analogous complex Cd···H_3_CH.^15^ Another initial vdW complex predicted here with CCSD(T), Zn···η^1^-HCH_3_**2a** of *C*_3v_ symmetry (Figure S1 of Supporting Information), has a somewhat lower binding energy than **2** (1.2 kJ/mol). No vdW complexes were reported in previous DFT studies of the Zn/CH_4_ system,^9,10^ which can be partially attributed to the well-known^16^ inability of conventional Kohn–Sham (KS) DFT to properly describe the dispersion forces. Complex **2** can, in principle, rearrange through insertion of the zinc atom into the C–H bond, which formally leads to the formation of the methylzinc hydride (HZnCH_3_(X^1^A_1_)) **3** species (Figure 1c). Before discussing the associated potential energy profile, we look more closely at the transition state involved.

**Table 2:** Relative Energies^a^ of the Stationary Points of the Reaction of Ground-State Atomic Zinc with Methane Calculated at the ZPVE-Corrected CCSD(T)/aug-cc-pV5Z and DK-CCSD(T)/aug-cc-pV5Z-DK Levels

species	aug-cc-pV5Z^b^	aug-cc-pV5Z-DK^b^,^c^
Zn(^1^S) + CH_4_ (**1**)	0.0	0.0
Zn···η^3^-H_3_CH (**2**)	–1.5	–1.5
Zn···η^1^-HCH_3_ (**2a**)^d^	–1.2	–1.2
**TS**	335.1	341.0
HZnCH_3_ (X^1^A_1_) (**3**)	45.2	47.7

a Relative to Zn(^1^S) + CH_4_ (**1**) (in kJ/mol).

b At the geometries optimized at the CCSD(T)/aug-cc-pVQZ level and including the CCSD(T)/aug-cc-pVTZ ZPVE contribution.

c Computed with the DK-CCSD(T) method.

d The optimized geometry of vdW complex **2a** is shown in Figure S1.

**Figure 2:**
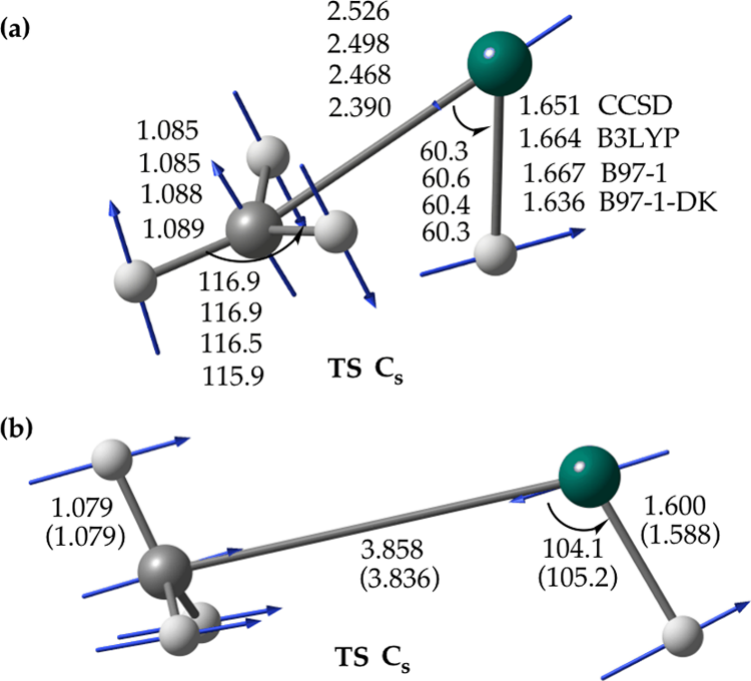
Comparison of **TS** structures for the reaction of ground-state atomic zinc with methane predicted with (a) B3LYP, B97-1, B97-1-DK KS DFT, and coupled-cluster CCSD using the aug-cc-pVTZ basis set and (b) coupled-cluster CCSD(T)/aug-cc-pVQZ method; structural parameters given in parentheses are from the DK-CCSD(T)/aug-cc-pVQZ-DK geometry optimization. Distances are in Å and angles are in degrees. Transition vectors corresponding to the imaginary frequency of the KS DFT **TS** (a) and CCSD(T) **TS** (b) structures are shown.

Nowadays, stationary points of the reaction of activation of methane by the small metal clusters are usually located using KS DFT, with the energetics possibly refined by CCSD(T).^17^ It is therefore interesting to compare the structures of the corresponding transition state (**TS**) of the reaction of ground-state atomic zinc with methane predicted using KS DFT with B97-1^18^ and B3LYP^19,20^ exchange–correlation functionals^21^ to that found using the coupled-cluster^22,23^ methods, an affordable task for the 3d-metal-containing-six-atom reaction system. These results show that *C*_s_-symmetric **TS** predicted by KS DFT (including B97-1-DK) and CCSD represents a “typical” transition state for oxidative insertion^9^ (Figure 2a, the C–H bond is lengthened to 2.12–2.23 Å, thus being essentially broken), with the corresponding imaginary frequency values ranging from 791*i* to 925*i* cm^–1^, and consistent with the transition state reported from the relativistic DFT ZORA-BLYP/TZ2P calculations.^9^ In contrast, the *C*_s_-symmetric **TS** found using CCSD(T) and DK-CCSD(T) (see Figure 2b), characterized by one imaginary frequency of 220*i* cm^–1^ (at the CCSD(T)/aug-cc-pVTZ level), is strongly asynchronous, featuring essentially a new Zn–H bond and a long Zn–C distance between ZnH and CH_3_ moieties of about 3.8 Å. The singlet CCSD(T) **TS** (Figure 2b) exhibits a significant multireference character^24^ and thus cannot be described correctly with the single-reference methods. We have not pursued this structure at the MR level because in the mechanism of HZnCH_3_(X^1^A_1_) formation considered here, the C–H activation step occurs through the relevant triplet transition state (as described in Section 2.4).

The ground-state singlet potential energy profile of the Zn + CH_4_ reaction calculated at the DK-CCSD(T)/aug-cc-pV5Z-DK//CCSD(T)/aug-cc-pVQZ level of theory (Table 2) indicates that (1) the process is endothermic by 47.7 kJ/mol, that is, by about 40 kJ/mol less than previously computed,^9,11^ and (2) it has an intractable energy barrier (of 341 kJ/mol), consistent with earlier reports;^9,11^ the **TS** barrier of similar magnitude is found here using MRPT2 (see below). The theoretical results are compatible with the experimental studies of the Zn + CH_4_ reaction^5−7^ wherein an external stimulus was always required to make the reaction happen, possibly in order to first induce the (4s^1^4p^1^)^3^P ← (4s^2^)^1^S electronic transition of zinc atoms to the reactive^5,7,25,26^ triplet state.

In Section 2.4, we will focus on the gas-phase activation of methane by excited ^3^P state atomic zinc. The spin–orbit coupling (SOC) (treated in Section 2.5) splits the ^3^P state of atomic zinc to the Zn(^3^P_*J*_) states (*J* = 0, 1, and 2), and it was the excitation of the ground-state zinc to the ^3^P_1_ state that caused insertion into the C–H bond of methane.^7^ The spin–orbit interaction results in a decrease in the energy of the Zn atom by 2.4 kJ/mol; the latter is the difference between the spin–orbit average value and the Zn ^3^P_1_ state.

### 2.3 Structure and Vibrational Frequencies of HZnCH_3_(X^1^A_1_)

Based on Figure 1c, the zinc insertion product HZnCH_3_(X^1^A_1_) **3** features a linear H–Zn–C backbone (*C*_3v_ symmetry), in agreement with the structure determined using rotational spectroscopy^5^ and quantum mechanical methods^8−11^ and consistent with the IR spectra of the matrix-isolated methylzinc hydride.^7^ It is also seen from this figure that there is good agreement between the equilibrium structure (*r*_e_) of **3** optimized at the DK-CCSD(T)/aug-cc-pVQZ-DK level (values in parentheses) and the *r*_o_ structure^5^ derived from measurements of the rotational spectra of the isotopologues of HZnCH_3_ (values in bold), in particular for the Zn–C and Zn–H bond lengths. For the C–H bond lengths, the agreement between the *r*_e_(C–H) (1.093 Å) and *r*_o_(C–H) (1.140 Å)^5^ values is less satisfactory; in fact, the former C–H bond distances are similar to those reported at the DFT level.^8−10^

Finally, in Table 3, the calculated harmonic (ω_i_) and anharmonic (ν_i_) vibrational frequencies of **3** are listed along with the available experimental data^7^ and previous^10^ harmonic DFT B3PW91 results. As this table indicates, the scalar relativistic effects cause an increase in the CCSD(T)/aug-cc-pVQZ harmonic frequencies of ZnH and ZnC stretch modes by 28 and 10 cm^–1^, respectively, due to an associated decrease in the two bond lengths (cf. Figure 1c). When the anharmonic contributions are considered by using second-order perturbation theory^28^ (see the values in the column of Table 3 under the heading “Hybrid”), the absolute deviation from the experiment is reduced to 18 cm^–1^ for the CH_3_ s-stretch (“s” stands for symmetric) and to 2–21 cm^–1^ for the next four modes with the lower frequencies. As a result, this leads to a better accordance with the experimental data^7^ compared to the previous DFT B3PW91 harmonic frequencies^10^ (the latter are given in the column of Table 3 under the heading “Literature”). A notable exception is the lowest frequency mode of **3**, CZnH a-deform (“a” stands for asymmetric) for which the largest difference (32 cm^–1^) between the “hybrid” and the experimental (harmonic DFT) values is observed.

**Table 3:** Harmonic (ω_i_) and Anharmonic (ν_i_) Vibrational Frequencies Calculated for the HZnCH_3_(X^1^A_1_) **3** Molecule along with the Experimental and Harmonic Literature Values (in cm^–1^)

		CCSD(T)/aug-cc-pVQZ	DK-CCSD(T)/aug-cc-pVQZ-DK	“Hybrid”^a^	literature^b^	
description of mode	sym. of vib.	ω_i_	ω_i_	ν_i_	ω_i_	exp.^c^
CH_3_ a-stretch	e	3103	3101	2960 (8)	3117	d
CH_3_ s-stretch	a_1_	3022	3016	2902 (9)	3037	2919.8
ZnH stretch	a_1_	1926	1954	1888 (212)	1901	1866.1
CH_3_ a-deform	e	1466	1453	1436 (0)	1452	d
CH_3_ s-deform	a_1_	1213	1213	1181 (3)	1203	1179.3
CH_3_ rock	e	700	684	666 (74)	712	686.8, 689.4
ZnC stretch	a_1_	568	577	569 (14)	562	566.5
CZnH a-deform	e	424	414	410 (46)	442	442.6

a Obtained in this work by combining the DK-CCSD(T)/aug-cc-pVQZ-DK harmonic vibrational frequencies with the anharmonic^28^ frequency corrections (based on the MP2/aug-cc-pVQZ calculations^29^); values in parentheses are the harmonic IR intensities (km/mol) obtained from the MP2/aug-cc-pVQZ calculations.

b Values in brackets are the DFT B3PW91 harmonic vibrational frequencies taken from ref (10).

c Data taken from the low-temperature matrix isolation IR study of the Zn/CH_4_ system reported in ref (7).

d Not observed in the matrix IR spectra.^7^

### 2.4 Activation of Methane by Excited ^3^P State Atomic Zinc

**Figure 3:**
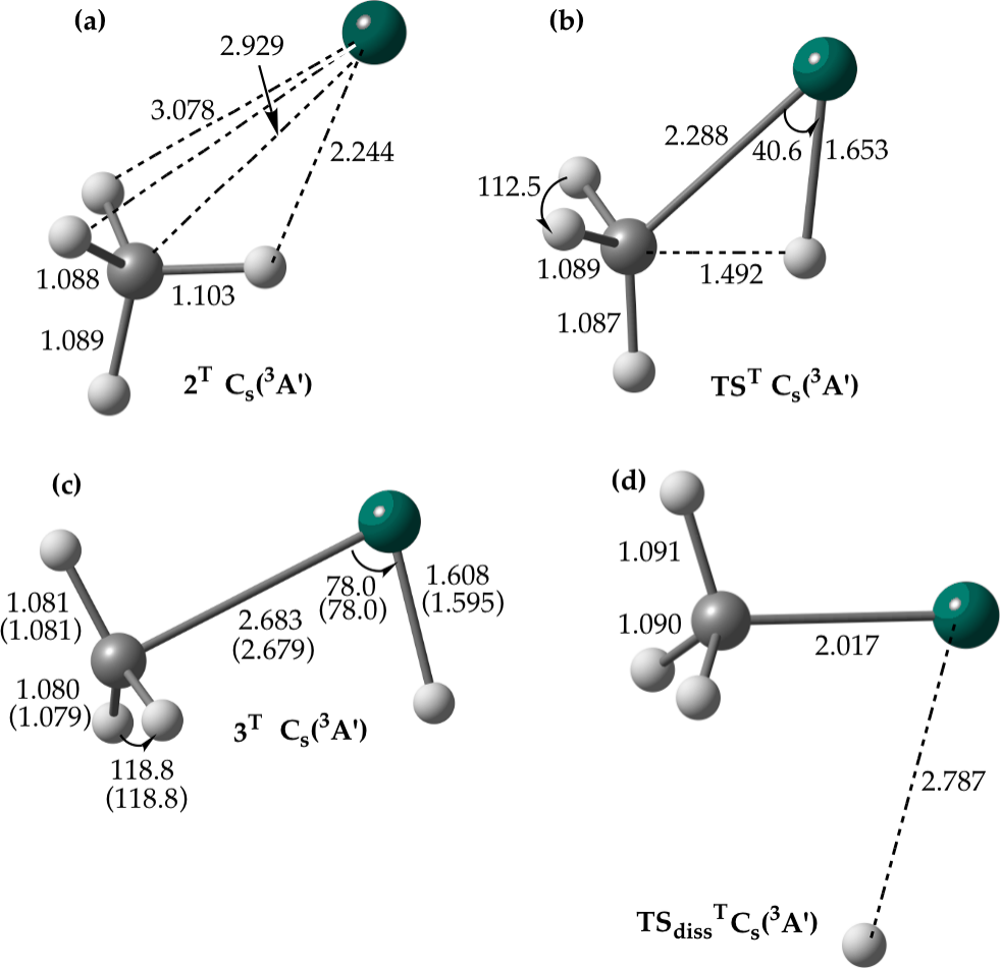
(a) “pre-activation” vdW complex Zn···η^1^-HCH_3_ (**2**^**T**^), (b) transition state for the C–H bond activation (**TS**^**T**^), (c) resulting intermediate HZnCH_3_ (**3**^**T**^), and (d) transition state for the H-atom dissociation (**TS**_**diss**_^**T**^) from **3**^**T**^, located on the lowest triplet state potential energy surface of the reaction of Zn(^3^P) with methane using the RCCSD(T)/aug-cc-pVQZ method. For **3**^**T**^, structural parameters shown in parentheses are from the DK-RCCSD(T)/aug-cc-pVQZ-DK geometry optimization. Due to the convergence problems in the numerical calculation of the nuclear hessian of **2**^**T**^ at the RCCSD(T)/aug-cc-pVnZ (n = D,T) levels, the actual hessian of **2**^**T**^ was computed analytically with the UMP2/aug-cc-pVTZ^29^ method (and found to be positive-definite). Distances are in Å and angles are in degrees.

The profile of the lowest triplet state potential energy surface of the Zn(^3^P) + CH_4_ reaction calculated at the DK-(R)CCSD(T)/aug-cc-pV5Z-DK//(R)CCSD(T)/aug-cc-pVQZ level of theory is shown in Table 4, with structures of the relevant species displayed in Figure 3. As observed above for the ground-state atomic zinc/methane reaction system, the reaction between Zn(^3^P) and methane involves formation of the initial vdW complex, Zn···η^1^-HCH_3_(^3^A′) **2**^**T**^ (Figure 3a, “T” stands for “Triplet”). In contrast, however, to the singlet counterpart **2** of *C*_3v_ symmetry, complex **2**^**T**^ (Figure 3a) adopts a *C*_s_ symmetry and exhibits a short Zn···H–C contact with the distance of 2.244 Å, indicating the occurrence of a Zn···H–C interaction similar to the agostic one.^30^ This interaction can be viewed as a pre-activation step as it is manifested by both the elongated C–H bond distance of 1.103 Å [the CCSD(T)/aug-cc-pVQZ (experimental^27^) bond length of methane is 1.088 (1.092 Å)] and the increase of the predicted binding energy of **2**^**T**^ in comparison to the singlet analogue **2**, 7.6 kJ/mol versus 1.5 kJ/mol, respectively, relative to the respective reactants [the relative energies of the triplet species quoted in this section are determined with respect to the Zn(^3^P_1_) + CH_4_ asymptote; cf. the values indicated in brackets in Table 4].

**Table 4:** Relative Energies^a^ of the Stationary Points of the Reaction of Excited ^3^P State Atomic Zinc with Methane Calculated at the ZPVE-Corrected (R)CCSD(T)/aug-cc-pV5Z and DK-(R)CCSD(T)/aug-cc-pV5Z-DK Levels.^b^

species^c^	aug-cc-pV5Z^d^	aug-cc-pV5Z-DK^d^^,^^e^^,^^f^
Zn(^3^P) + CH_4_ (**1**)	371.5 (0.0)	389.9 (0.0) [0.0]
Zn···η^1^-HCH_3_ (**2**^**T**^)	362.3 (−9.4)	379.9 (-10.0) [−7.6]
**TS**^**T**^(^3^A′)	397.5 (25.9)	409.2 (19.2) [21.6]
HZnCH_3_(^3^A′) (**3^T^**)	340.2 (−31.4)	346.0 (−43.5) [−41.1]
**TS**_**diss**_^**T**^(^3^A′)	369.4 (−2.1)	374.9 (−14.6) [-12.2]
ZnH(^2^Σ^+^) + CH_3_(^2^A_2_^″^)	343.5 (−28.0)	349.4 (−40.6) [−38.2]
ZnCH_3_(^2^A_1_) + H(^2^S)	366.9 (−4.6)	372.8 (−17.2) [−14.8]

a Relative to Zn(^1^S) + CH_4_ (**1**) except for the energies indicated in parentheses which are relative to Zn(^3^P) + CH_4_ (**1**) (in kJ/mol).

b For the open-shell species, this refers to the corresponding RCCSD(T) levels.

c For the optimized geometries of the ZnCH_3_(^2^A_1_), ZnH(^2^Σ^+^), and CH_3_(^2^A_2_^″^) radicals, see Figure S5.

d At the geometries optimized at the CCSD(T)/aug-cc-pVQZ level and including the CCSD(T)/aug-cc-pVTZ ZPVE contribution.

e Computed with the DK-CCSD(T) method.

f The energies (in kJ/mol) indicated in brackets are also corrected for spin–orbit effects.

On the triplet reaction potential surface, the C–H bond activation is found to be exothermic by 41.1 kJ/mol with respect to Zn(^3^P_1_) + CH_4_ (Table 4) and proceeds from **2^T^** to the intermediate **3^T^**(^3^A′) (Figure 3c) via the “early” transition state **TS^T^**(^3^A′) (Figure 3b), the latter characterized by one imaginary frequency of 1332*i* cm^–1^ (at the RCCSD(T)/aug-cc-pVTZ level). Note that in the **TS^T^** → **3^T^** step, the CH_3_ group rotates about the Zn–C axis (as confirmed by the IRC following using the UMP2/aug-cc-pVTZ method^29^). Interestingly, the corresponding energy barrier of 21.6 kJ/mol [with respect to Zn(^3^P_1_) + CH_4_], derived from the DK-(R)CCSD(T)/aug-cc-pV5Z-DK calculations (Table 4), shows good agreement with the experimental activation energy of the Zn(^3^P_1_) + CH_4_ process (∼14.7 kJ/mol) inferred from the matrix-isolation IR study of the radiation-induced reaction of zinc atoms with methane of Downs and co-workers^7^ (the estimate referred to therein as an “approximate upper limit”^7^). The latter two barrier evaluations are at variance with that of a previous computational study of the Zn(^3^P) + CH_4_ reaction by Castillo et al.^11^ who reported a much higher energy barrier (75.3 kJ/mol).

Compared to the singlet **3**, the triplet **3**^**T**^ differs vastly in the equilibrium geometry and the depth of the corresponding potential well, congruent with the DFT study of Alikhani.^10^ Namely, **3**^**T**^ (Figure 3c) features a bent H–Zn–C backbone with the acute H–Zn–C bond angle of 78.0° and the Zn–C distance being longer than that of **3** by as much as 0.75 Å (at the DK-RCCSD(T)/aug-cc-pVQZ-DK level). Consequently, **3**^**T**^ is predicted to be only marginally stable (by 2.9 kJ/mol) with respect to dissociation into ZnH(^2^Σ^+^) + CH_3_(^2^A_2_^″^) (Table 4), and, therefore, it can be represented as HZn···CH_3_(^3^A′); there is no energy barrier above the dissociation energy, consistent with an earlier report.^11^ By examining a higher energy dissociation channel of **3**^**T**^ into ZnCH_3_(^2^A_1_) + H(^2^S), we have found that it takes place via the **TS**_**diss**_^**T**^(^3^A′) transition state (Figure 3d) that lies 28.9 kJ/mol above **3**^**T**^, giving rise to the energy barrier of 2.6 kJ/mol beyond the dissociation energy (Table 4).

### 2.5 Plausible Mechanism for the Formation of HZnCH_3_(X^1^A_1_) in the Gas Phase Involving Zn(^3^P) and CH_4_. Is This a Viable Reaction Pathway?

Under the experimental conditions used in the gas-phase study,^5^ the reactant mixture containing zinc vapor (Zn(g)) and methane was exposed to a DC discharge, with the latter described^5^ as “not a selective excitation method”, meaning that the Zn (4s^1^4p^1^)^3^P ← (4s^2^)^1^S and (4s^1^4p^1^)^1^P ← (4s^2^)^1^S electronic transitions could have occurred. In this section, a plausible scenario for the formation of HZnCH_3_(X^1^A_1_) **3** is considered that involves activation of methane by excited ^3^P state atomic zinc [note that the activation of CH_4_ by excited Zn(^1^P) was the subject of a previous theoretical study^11^].

**Figure 4:**
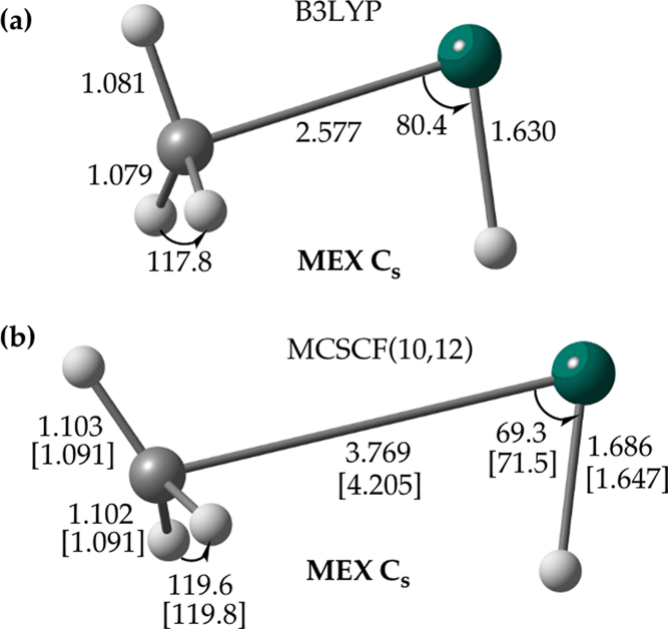
**MEX** between the lowest singlet (^1^A′) and the lowest triplet (^3^A′) state potential energy surfaces of the Zn + CH_4_ reaction determined with (a) B3LYP using the aug-cc-pVTZ basis set and (b) MCSCF(10,12) using the def2-SVP and aug-cc-pVTZ basis sets; the structural parameters obtained with the latter basis set are given in brackets. The B3LYP/aug-cc-pVTZ and MCSCF/aug-cc-pVTZ **MEX** structures have been located with GAMESS, and the MCSCF/def2-SVP one has been found with MOLPRO. Distances are in Å and angles are in degrees.

We looked first at the HZnCH_3_(X^1^A_1_) formation pathway that involves intersystem crossing. Using both coupled-perturbed MCSCF(10,12)/def2-SVP^31^ (implemented in MOLPRO^32^) and MCSCF(10,12)/aug-cc-pVTZ combined with the constrained optimization method^33^ (the latter implemented in GAMESS^34^), the minimum energy crossing point (**MEX**) between the lowest triplet and singlet electronic states has been located in the vicinity of the triplet intermediate **3**^**T**^ (Figure 4b). The qualitatively similar type of **MEX** has been found at the B3LYP/aug-cc-pVTZ level using GAMESS^33,34^ (Figure 4a), consistent with a previous DFT^10^ report. The major difference between the two **MEX** structures is the significantly longer Zn–C distance predicted with MCSCF compared to that found with B3LYP. This is due to insufficient dynamic correlation in the MCSCF method, which has implications for the location of the system’s **MEX**(**s**).^35^ The rate of intersystem crossing is known to depend on the SOC.^36^ To compute the SOC matrix element at the **MEX**, we first applied the approximate one-electron Breit–Pauli spin–orbit operator combined with the effective nuclear charge approach^37^ along with state-averaged MCSCF wave functions employing the relativistic Stevens–Basch–Krauss–Jasien–Cundari (SBKJC) effective core potentials and associated basis sets (MCSCF/SBKJC ECP).^37^ In addition, the Breit–Pauli spin–orbit operator including the one-electron and two-electron terms was used in the interacting-states approach^38^ with state-averaged MCSCF wave functions and for the purpose of testing, with MRCI^39,40^ wave functions. The performance of these various SOC computing procedures was evaluated through calculation of the spin–orbit splittings in the ^3^P state of atomic zinc (Table 5), which shows that the MCSCF(2,4)/SBKJC ECP and DK-MCSCF(2,4)/aug-cc-pVTZ-DK (cc-pVTZ-DK) results are in reasonable agreement with those obtained by the DK-MRCI(2,4)/aug-cc-pwCVTZ-DK (cc-pwCVTZ-DK) approach and experiment.^12^ The magnitude of the calculated SOC constant^36^ at the **MEX** is strongly dependent on the Zn–C distance. That is, at the MCSCF geometry, the SOC constant is found to be in the range of 1.05–1.83 cm^–1^ with the MCSCF(2,4)/SBKJC ECP and DK-MCSCF(2,4)/aug-cc-pVTZ-DK procedures and at the B3LYP geometry, it is predicted to be 28.56 cm^–1^ (MCSCF(2,4)/SBKJC ECP) and 38.73 cm^–1^ (DK-MCSCF(2,4)/aug-cc-pVTZ-DK). Recall that **MEX** actually constitutes the HZn···CH_3_ radical pair and for the radical pairs, SOC is known to decrease sharply as the separation of the radical centers increases.^41^

**Table 5:** Spin–Orbit Splitting for Zn(^3^P) (in cm^–1^)

method	|^3^P_0_–^3^P_1_|	|^3^P_1_–^3^P_2_|
MCSCF(2,4)/SBKJC ECP^a^	180.20	360.43
DK-MCSCF(2,4)/aug-cc-pVTZ-DK^b^^,^^c^	165.93 (167.13)	331.87 (334.27)
DK-MRCI(2,4)/aug-cc-pVTZ-DK^c^^,^^d^	170.98 (171.22)	341.95 (342.45)
DK-MRCI(2,4)/aug-cc-pwCVTZ-DK^e^^,^^f^	188.01 (183.76)	376.01 (367.53)
exp.^g^	190.08	388.93

a Computed with the state-averaged MCSCF method using SBKJC ECPs and an approximate one-electron Breit–Pauli spin–orbit operator combined with the effective nuclear charge (*Z*_eff_) approach; see ref (37) for the relevant references.

b Computed with the state-averaged MCSCF method and Breit–Pauli spin–orbit operator (including the one-electron and two-electron terms) in the interacting-states approach.^38^

c Values in parentheses are obtained with the cc-pVTZ-DK basis set.

d Only valence electrons correlated in the MRCI calculations (see also footnote^b^).

e All electrons correlated in the MRCI calculations (see also footnote^b^).

f Values in parentheses are obtained with the cc-pwCVTZ-DK basis set.

g Taken from Moore’s tables.^12^

Table 6 shows the MRPT2 energies of the species relevant to the formation and dissociation of HZnCH_3_(X^1^A_1_) **3**. As can be seen, there is an overall good agreement between the predictions of the two MRPT2 methods. In addition, significant scalar relativistic effects are found by comparing the CASPT2/aug-cc-pVTZ and DK-CASPT2/aug-cc-pVTZ-DK energies (ranging from 4.6 to 18 kJ/mol), in line with the coupled-cluster calculations.

**Table 6:** MRPT2 Relative Energies^a^^,^^b^ of the Species Relevant to the Formation and Dissociation of HZnCH_3_(X^1^A_1_) **3** (in kJ/mol)

species	MCQDPT2(10,12)/aug-cc-pVTZ	CASPT2(10,12)/aug-cc-pVTZ	DK-CASPT2(10,12)/aug-cc-pVTZ-DK
Zn(^3^P) + CH_4_ (**1**)	366.1 (0.0)	364.0 (0.0)	382.0 (0.0)
**TS**^**T**^(^3^A′)	395.4 (29.3)	397.9 (33.9)	408.8 (26.4)
HZnCH_3_(^3^A′) (**3**^**T**^)	356.5 (−9.6)	355.2 (−8.8)	361.5 (−20.5)
**MEX**	356.5 (−9.6)	356.1 (−7.9)	362.3 (−19.7)
ZnH(^2^Σ^+^) + CH_3_(^2^A_2_^″^)	370.7 (4.6)	363.2 (−0.8)	369.0 (−13.0)
ZnCH_3_(^2^A_1_) + H(^2^S)	380.7 (14.6)	381.6 (17.6)	387.0 (5.0)
HZnCH_3_ (X^1^A_1_) (**3**)	46.9 (−319.2)	46.4 (−317.6)	50.6 (−331.4)

a With respect to Zn(^1^S) + CH_4_ (**1**), the energies indicated in parentheses are relative to Zn(^3^P) + CH_4_ (**1**). Notice that these energies do not include corrections for ZPVE and spin–orbit effects (because **MEX** is not a stationary point on the 3N-6-dimensional potential energy surface, the frequency calculations could not be performed in this case). At the three consecutive MRPT2 levels indicated, the energies of **TS** (assuming the CCSD(T)/aug-cc-pVTZ geometry) are 355.2 (−10.9), 354.8 (−9.2), and 359.8 (−22.2) kJ/mol, respectively.

b At the geometries optimized at the MCSCF(10,12)/aug-cc-pVTZ level.

**Figure 5:**
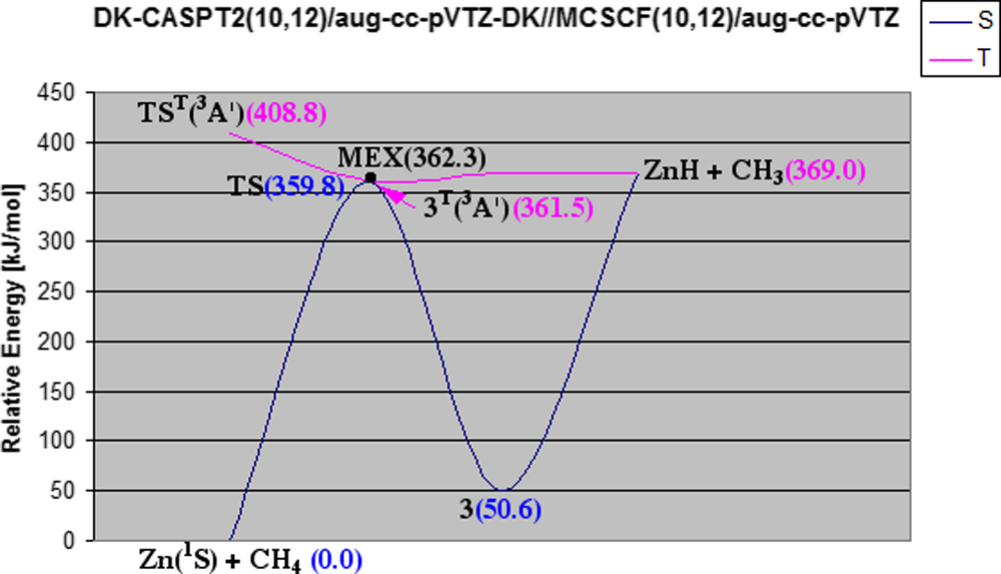
Relative energies of the key stationary points on the lowest singlet and triplet potential surfaces of the Zn + CH_4_ reaction relevant to the formation of HZnCH_3_(X^1^A_1_) **3** as calculated at the DK-CASPT2(10,12)/aug-cc-pVTZ-DK//MCSCF(10,12)/aug-cc-pVTZ level (note that these energies do not include corrections for ZPVE and spin–orbit effects). Singlet (S) potential surface, navy blue; triplet (T) potential surface, pink; and **MEX** is denoted by the black dot. The values in parentheses are in kJ/mol.

Next, we considered a somewhat different mechanistic scenario of the formation of HZnCH_3_(X^1^A_1_) **3** involving the Zn(^3^P) and CH_4_ reactants, viewed to be a more plausible mechanism^42^ in the gas phase. In addition to the Zn ^3^P ← ^1^S electronic excitation and activation of methane by excited zinc through the **TS**^**T**^ transition state to yield **3**^**T**^, this scenario allows the dissociation of the triplet intermediate HZn···CH_3_(^3^A′) **3**^**T**^ into ZnH(^2^Σ^+^) and CH_3_(^2^A_2_^″^) and involves the recombination of the resulting radicals followed by relaxation to **3** (Figure 5). This scenario corresponds basically to a radical rebound mechanism^43^ that does not involve **MEX**. The presence of a “third body” Ar in the reaction chamber^5^ could have provided a stabilization of the HZnCH_3_(X^1^A_1_) **3** product (as it was also mentioned by Ziurys and co-workers^5^). Although some experimental evidence in favor of a radical mechanism for the formation of HZnCH_3_(X^1^A_1_) has been provided because “weak ZnH signals” were detected,^5^ a reaction dynamics investigation is needed to further support this mechanism.^43^

Because, as disclosed above, both excited ^3^P and ^1^P state atomic zinc could have been produced under the experimental conditions employed in the gas-phase study,^5^ the zinc atom in the ^1^P excited state could have contributed to the C–H bond activation as well.^42^ That was spectroscopically observed in low-temperature matrix studies of the reaction of Zn(^1^P) with methane.^8^

## 3 Conclusions

We have probed potential energy surfaces of the reaction of ground ^1^S and excited ^3^P state atomic zinc with methane [using the CCSD(T) method] along with intersystem crossing (studied with MCSCF/MRPT2) and the importance of the scalar relativistic effects (included via the second-order DK method) on the reaction’s energetics and the structure of the methylzinc hydride (HZnCH_3_(X^1^A_1_)) product molecule. The latter molecule was previously^5^ synthesized in the gas phase in a DC discharge by the reaction of zinc vapor with methane and observed using rotational spectroscopy. The main results are as follows.

(1) The reaction of ground-state atomic zinc and methane to afford the methylzinc hydride (HZnCH_3_(X^1^A_1_)) is predicted to be endothermic relative to the separated reactants (by 48 kJ/mol) and requires overcoming an intractable energy barrier (of 340 kJ/mol), these results being compatible with the experimental studies^5−7^ and earlier calculations.^9,11^ There is good agreement between the equilibrium geometry (*r*_e_) of the HZnCH_3_(X^1^A_1_) molecule predicted with the DK-CCSD(T)/aug-cc-pVQZ-DK method and the corresponding *r*_o_ structure derived^5^ from rotational spectroscopy, especially for the Zn–C and Zn–H bond lengths.
(2) The reaction of excited ^3^P state atomic zinc with methane entails the formation of a “pre-activation” vdW complex that exhibits agostic-like Zn···H–C interaction. Involvement of Zn(^3^P) in the gas-phase reaction with methane has led to a dramatic decrease in the activation barrier compared to that found for ground-state atomic zinc. Moreover, the magnitude of the energy barrier of 21.6 kJ/mol as determined at the ZPVE and spin–orbit-corrected DK-(R)CCSD(T)/aug-cc-pV5Z-DK//(R)CCSD(T)/aug-cc-pVQZ level of theory is supported by the experimental activation energy of the Zn(^3^P_1_) + CH_4_ reaction (∼14.7 kJ/mol) inferred from the matrix-isolation IR study of the radiation-induced reaction of zinc atoms with methane.^7^ Significant scalar relativistic effects on the exothermicity of the overall Zn(^3^P) + CH_4_ → HZnCH_3_ (^3^A′) process and related energy barrier are found, that is, an increase by 12.1 kJ/mol and a decrease by 6.7 kJ/mol, respectively.
(3) A plausible mechanism for the formation of HZnCH_3_(X^1^A_1_) in the gas phase^5^ is suggested, which involves activation of the C–H bond of methane by the lowest excited ^3^P state atomic zinc to give the loosely bound intermediate HZn···CH_3_(^3^A′), which dissociates into ZnH and CH_3_, followed by the recombination of the resulting radicals. A reaction dynamics investigation is necessary to provide further support of the mechanism proposed.

## 4 Computational Methods

Potential energy surfaces of the reaction of atomic zinc with methane were probed using CCSD(T)^23^ in conjunction with the aug-cc-pVnZ (n = D,T,Q) basis sets.^44−47^ For the open-shell species, the spin-restricted CCSD(T) method,^48^ denoted as RCCSD(T), was employed. The relevant stationary points were confirmed by harmonic vibrational frequency analyses carried out at the (R)CCSD(T)/aug-cc-pVnZ levels with n = D,T. Single-point (R)CCSD(T)/aug-cc-pV5Z calculations were also reported. The scalar relativistic effects were considered using the second-order Douglas–Kroll–Hess (DK) Hamiltonian^49,50^ in combination with the aug-cc-pVnZ-DK (n = T,Q,5) basis sets.^44−47,51^ In all of the coupled-cluster calculations, Zn 1s2s2p3s3p and C 1s atomic orbitals were kept frozen (not correlated). Only the (R)CCSD(T)/aug-cc-pVQZ (and DK-(R)CCSD(T)/aug-cc-pVQZ-DK) structures have been presented in the main text (for a comparison of the (R)CCSD(T)/aug-cc-pVnZ structures with n = D,T,Q, see Figures S2, S8 and S9 of the Supporting Information).

The multireference treatment was accomplished by MCSCF wave function calculations of the complete active space (CAS)^52,53^ or fully optimized reaction space^54^ type, with the active space consisting of 10 electrons in 12 orbitals, (10,12), arising from Zn 4s4p, C 2s2p, and H 1s orbitals, followed by calculations using second-order multiconfiguration quasi-degenerate perturbation theory (MCQDPT2)^55^ and CASPT2^56^ implementation of MRPT2 to account for dynamic correlation effects (when applied to one state only, MCQDPT2 is equivalent to multireference second-order Møller–Plesset perturbation theory, MRMP2^57,58^).

MOLPRO2012.1^32^ quantum chemistry code was used for the CC and CASPT2 computations and GAMESS^34^ code was exploited for the MCQDPT2 computations. Both programs were employed for the spin–orbit computations. The MP2 (UMP2) and KS DFT calculations were performed with the GAUSSIAN16^59^ program (more computational details are provided in the Supporting Information).

## Supporting Information Available

Full citation for refs (32, 34, 59), additional vdW complex Zn···η^1^-HCH_3_**2a** formed due to interaction between ground-state atomic zinc and methane as optimized at the CCSD(T)/aug-cc-pVnZ (n = D,T,Q) levels; “full version” of Figure 3; third-order saddle point **TOSP**_**abst**_^**T**^ located on the lowest triplet state potential energy surface of the reaction of Zn(^3^P) with methane using the RCCSD(T)/aug-cc-pVnZ (n = D,T) methods; structures of the triplet intermediate HZnCH_3_(^3^A′) **3**^**T**^ optimized at the MCSCF(10,12)/aug-cc-pVTZ, MCSCF(10,12)/def2-SVP, and RB3LYP/aug-cc-pVTZ levels; structures of the ZnCH_3_(^2^A_1_), CH_3_(^2^A_2_^″^), and ZnH(^2^Σ^+^) radicals optimized at the RCCSD(T)/aug-cc-pVnZ (n = D,T,Q) levels; vdW complex Zn···η^1^-HCH_3_**2**^**T**^ located on the lowest triplet state potential energy surface of the reaction of Zn(^3^P) with methane using the UMP2/aug-cc-pVTZ method; structures of HZnCH_3_(X^1^A_1_) **3** and transition state **TS**^**T**^(^3^A′) for the C–H bond activation optimized at the MCSCF(10,12)/aug-cc-pVTZ level; “full versions” of Figures 1 and 2 and of Tables 1, 2 and 4; and “Additional Computational Details” section (PDF)
