# Supplementary material for: C–H Bond Activation by the Excited Zinc Atom: Gas-Phase Formation of Methylzinc Hydride (HZnCH3) Based on Multireference Second-Order Perturbation Theory and Coupled Cluster Calculations
Source: ACS Omega. 2021 Sep 6;6(37):24280–8. doi: 10.1021/acsomega.1c04531 (PMC8459409; doi:10.1021/acsomega.1c04531)
Supplement: Supplementary file 1 — ao1c04531_si_001.pdf [file ao1c04531_si_001.pdf]

Supporting Information
to
C-H Bond Activation by the Excited Zinc Atom: Gas-Phase Formation of Methylzinc Hydride ($HZnCH_3$) Based on Multireference Second-Order Perturbation Theory and Coupled Cluster Calculations

Jerzy Moc*

Faculty of Chemistry, Wroclaw University, F. Joliot-Curie 14, 50-383 Wroclaw, Poland

*Correspondence should be addressed to J.M. (*jerzy.moc@chem.uni.wroc.pl*)

Table of Content

1. Full references 32, 34 and 59
2. Figures S1-S9
3. Tables S1-S3
4. Additional Computational Details

1. Full reference 32.

MOLPRO: Werner, H.-J.; Knowles, P. J.; Knizia, G.; Manby, F. R.; Schütz, M.; Celani, P.; Korona, T.; Lindh, R.; Mitrushenkov, A.; Rauhut, G.; Shamasundar, K. R.; Adler, T. B.; Amos, R. D.; Bernhardsson, A.; Berning, A.; Cooper, D. L.; Deegan, M. J. O.; Dobbyn, A. J.; Eckert, F.; Goll, E.; Hampel, C.; Hesselmann, A.; Hetzer, G.; Hrenar, T.; Jansen, G.; Köppl, C.; Liu, Y.; Lloyd, A. W.; Mata, R. A.; May, A. J.; McNicholas, S. J.; Meyer, W.; Mura, M. E.; Nicklass, A.; O'Neill, D. P.; Palmieri, P.; Peng, D.; Pflüger, K.; Pitzer, R.; Reiher, M.; Shiozaki, T.; Stoll, H.; Stone, A. J.; Tarroni, R.; Thorsteinsson, T.; Wang, M. *MOLPRO*, version 2012.1; Cardiff University: Cardiff, U.K., 2012.

Full reference 34.

GAMESS: Schmidt, M. W.; Baldridge, K. K.; Boatz, J. A.; Elbert, S. T.; Gordon, M. S.; Jensen, J. H.; Koseki, S.; Matsunaga, N.; Nguyen, K. A.; Su, S.; Windus, T. L.; Dupuis, M.; Montgomery Jr. J. A. General Atomic and Molecular Electronic Structure System. *J. Comput. Chem.* **1993**, *14*, 1347-1363.

Full reference 59.

Gaussian 16: Frisch, M. J.; Trucks, G. W.; Schlegel, H. B.; Scuseria, G. E.; Robb, M. A.; Cheeseman, J. R.; Scalmani, G.; Barone, V.; Petersson, G. A.; Nakatsuji, H.; Li, X.; Caricato, M.; Marenich, A. V.; Bloino, J.; Janesko, B. G.; Gomperts, R.; Mennucci, B.; Hratchian, H. P.; Ortiz, J. V.; Izmaylov, A. F.; Sonnenberg, J. L.; Williams-Young, D.; Ding, F.; Lipparini, F.; Egidi, F.; Goings, J.; Peng, B.; Petrone, A.; Henderson, T.; Ranasinghe, D.; Zakrzewski, V. G.; Gao, J.; Rega, N.; Zheng, G.; Liang, W.; Hada, M.; Ehara, M.; Toyota, K.; Fukuda, R.; Hasegawa, J.; Ishida, M.; Nakajima, T.; Honda, Y.; Kitao, O.; Nakai, H.; Vreven, T.; Throssell, K.; Montgomery, J. A., Jr.; Peralta, J. E.; Ogliaro, F.; Bearpark, M. J.; Heyd, J. J.; Brothers, E. N.; Kudin, K. N.; Staroverov, V. N.; Keith, T. A.; Kobayashi, R.; Normand, J.; Raghavachari, K.; Rendell, A. P.; Burant, J. C.; Iyengar, S. S.; Tomasi, J.; Cossi, M.; Millam, J. M.; Klene, M.; Adamo, C.; Cammi, R.; Ochterski, J. W.; Martin, R. L.; Morokuma, K.; Farkas, O.; Foresman, J. B.; Fox, D. J. *Gaussian 16, Revision C.01*, Gaussian, Inc., Wallingford CT, 2016.

2. Figures S1-S9

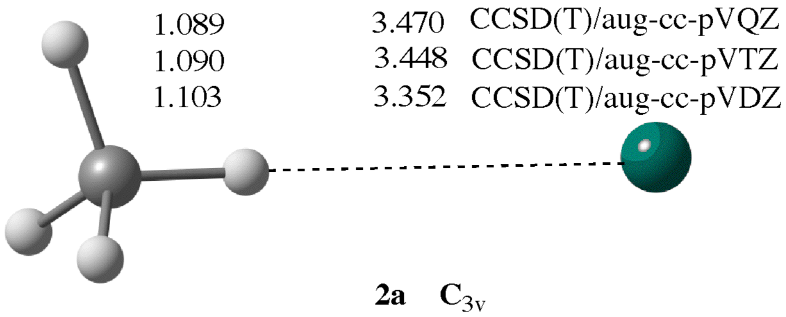

2a C_{3v}

Figure S1.

Figure S1. Alternative vdW complex Zn**…**HCH$_3$ (**2a**) for the reaction of ground-state atomic zinc with methane optimized at the CCSD(T)/aug-cc-pV*n*Z (*n*=D,T,Q) levels. Distances are in Å.

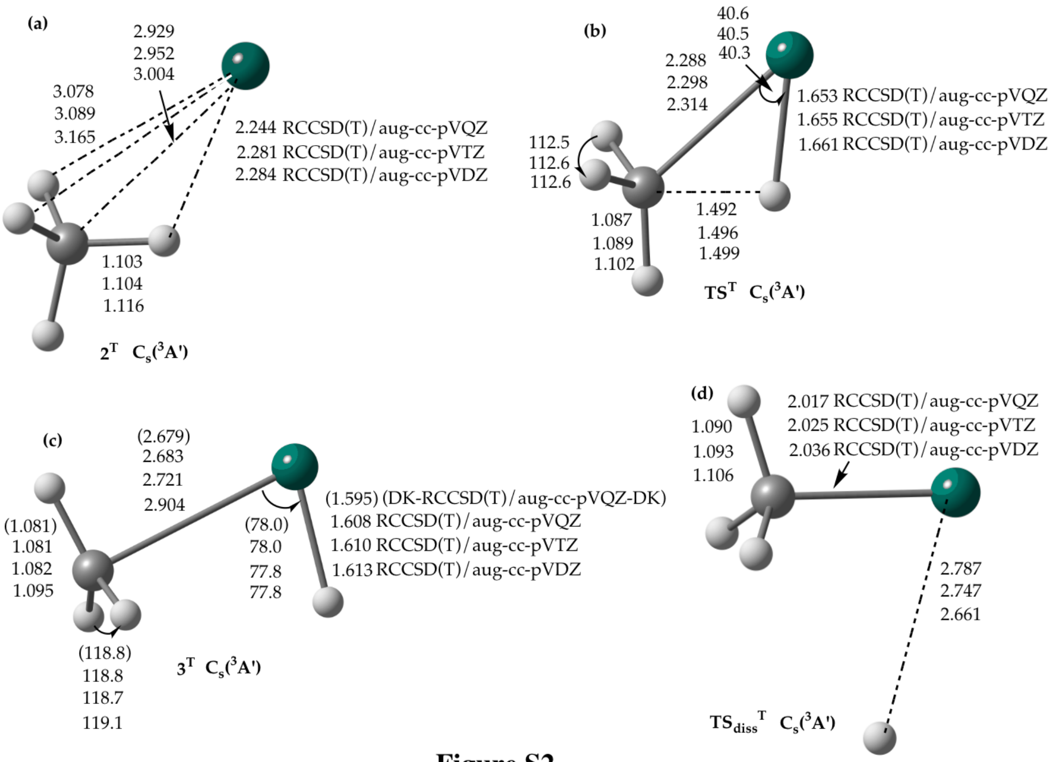

Figure S2.

Figure S2. Structures of (a) vdW complex (**2**^{T}), (b) transition state for the C-H bond activation (**TS**^{T}), (c) resulting intermediate $HZnCH_3$ (**3**^{T}), and (d) transition state for H dissociation (**TS**$_{diss}^{T}$) from **3**^{T} located on the lowest triplet state potential energy surface of the Zn(^{3}P) + CH_4 reaction using the RCCSD(T)/aug-cc-pV*n*Z (*n*=D,T,Q) methods. For **3**^{T}, structural parameters shown in parentheses are from the DK-RCCSD(T)/aug-cc-pVQZ-DK geometry optimization. Distances are in Å, and angles are in degrees.

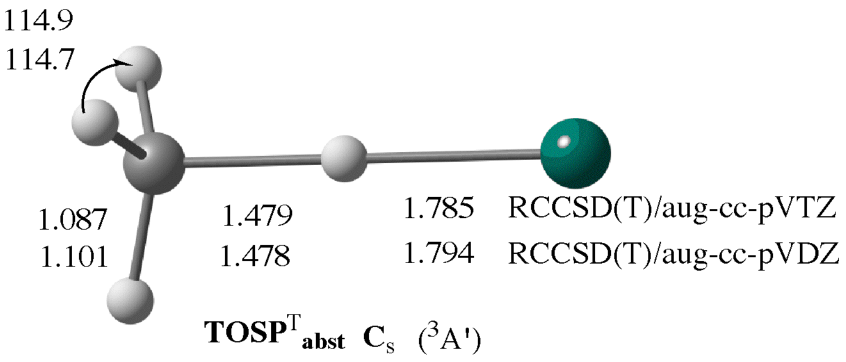

Figure S3.

Figure S3. A search for the transition state in the abstraction mechanism of the Zn(^{3}P) + CH_4 reaction has led to the stationary-point structure of C_s (^{3}A′) symmetry that appeared to be the third-order saddle point, **TOSP^T$_{abst}$**; in addition to an imaginary frequency for the correct abstraction mode, it produced an imaginary frequency corresponding to the degenerate bending mode. If one followed the latter mode, **TOSP^T$_{abst}$** eventually optimized to **TS^T**.

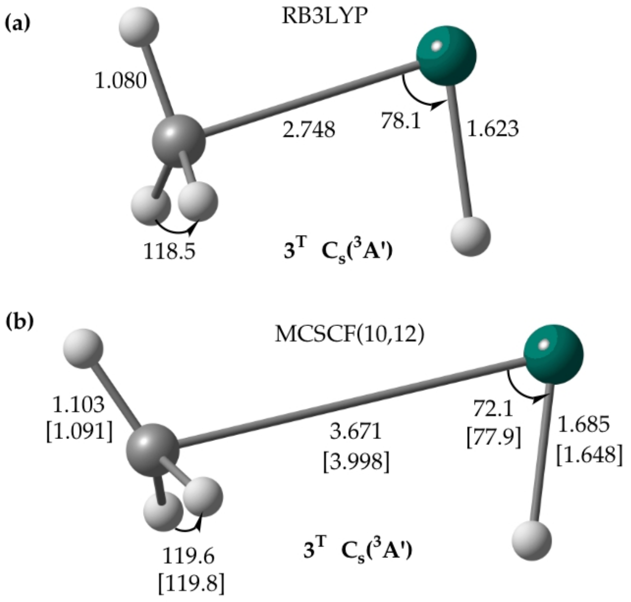

Figure S4.

Figure S4. Comparison of structures of the triplet intermediate $HZnCH_3$ (**3**^{T}) optimized with: (a) RB3LYP KS DFT using the aug-cc-pVTZ basis set, and (b) MCSCF(10,12) using the def2-SVP and aug-cc-pVTZ basis sets (the structural parameters obtained with the latter basis set are given in brackets). Distances are in Å, and angles are in degrees.

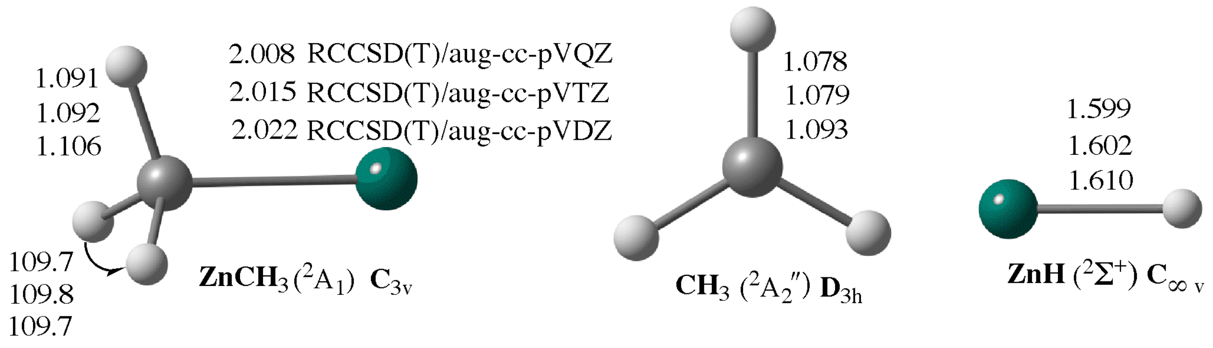

Figure S5.

Figure S5. Structures of the $ZnCH_3$, CH_3, and ZnH radicals in their ground electronic states optimized at the RCCSD(T)/aug-cc-pV*n*Z (*n*=D,T,Q) levels. Distances are in Å, and angles are in degrees.

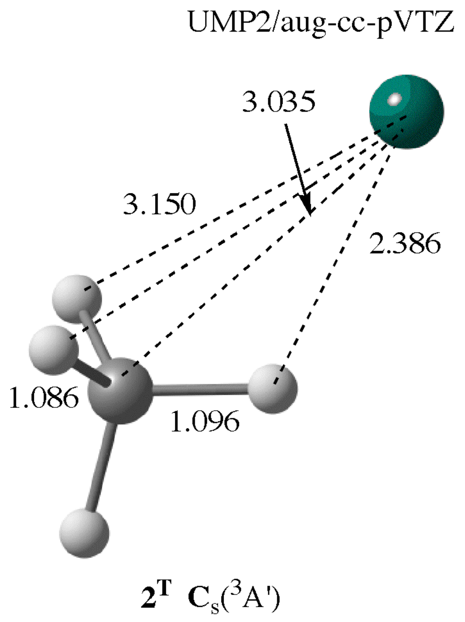

Figure S6.

Figure S6. Initial vdW complex $\mathbf{2^T}$ located on the lowest triplet state potential energy surface of the Zn(^{3}P) + CH_4 reaction using the UMP2/aug-cc-pVTZ method. Distances are in Å.

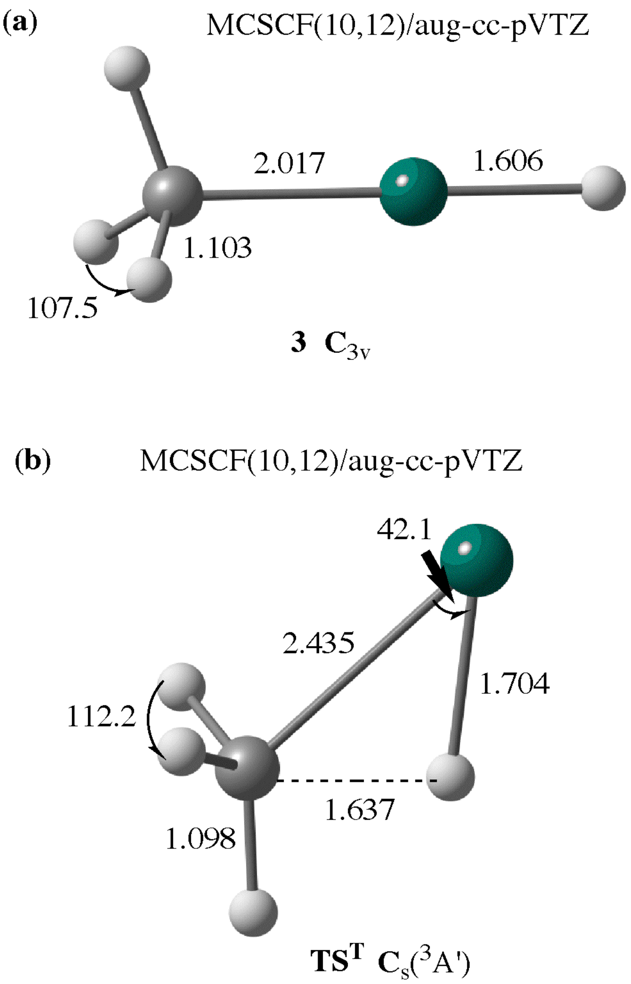

Figure S7.

Figure S7. (a) Structure of $HZnCH_3(X^1A_1)$ (**3**) optimized at the MCSCF(10,12)/aug-cc-pVTZ level; (b) Structure of transition state for the C-H bond activation (**TS^T**) located on the lowest triplet state potential energy surface of the $Zn(^3P) + CH_4$ reaction optimized at the MCSCF(10,12)/aug-cc-pVTZ level. Bond distances are in Å, and bond angles are in degrees.

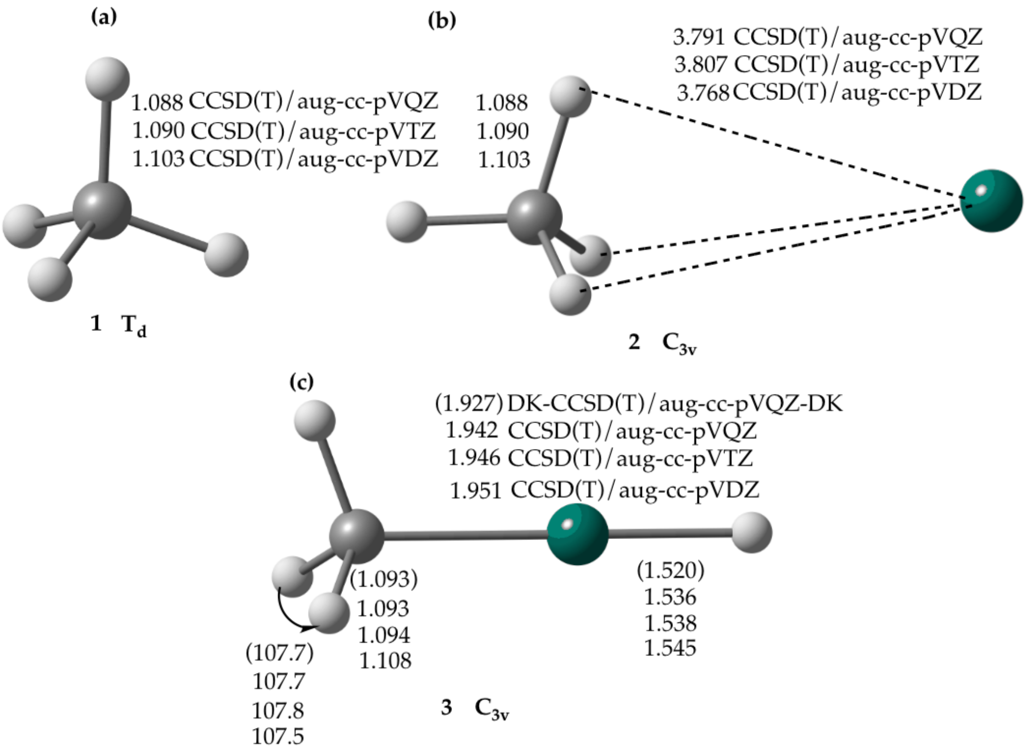

Figure S8.

Figure S8. Structures of (a) reactant CH_4 (**1**), (b) vdW complex Zn**…**H_3CH (**2**), and (c) insertion product $HZnCH_3(X^1A_1)$ (**3**) of the reaction of ground-state atomic zinc with methane optimized at the CCSD(T)/aug-cc-pV*n*Z (*n*=D,T,Q) levels. For **3**, structural parameters shown in parentheses are from the DK-CCSD(T)/aug-cc-pVQZ-DK geometry optimization. Distances are in Å.

(a)

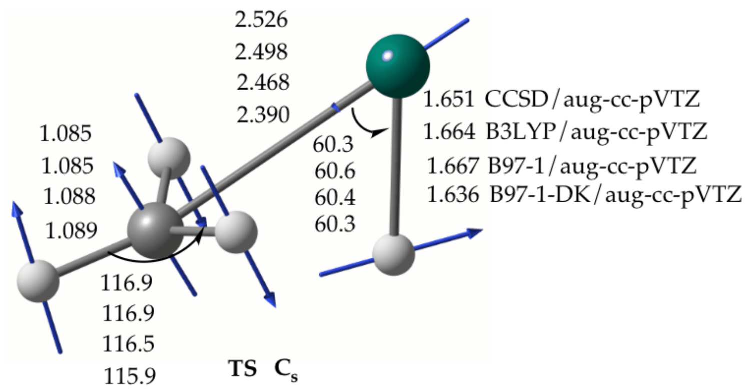

(b)

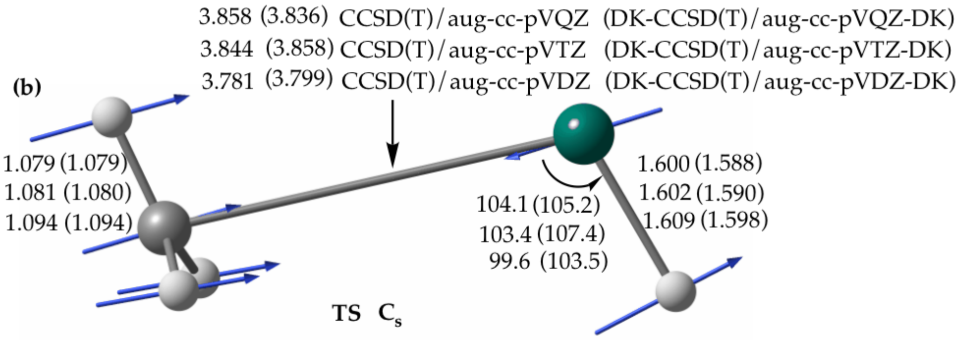

Figure S9.

Figure S9. Comparison of transition state (**TS**) structures for the reaction of ground-state atomic zinc with methane predicted with: (a) B3LYP, B97-1, B971-DK KS DFT and coupled-cluster CCSD using the aug-cc-pVTZ basis set, (b) coupled-cluster CCSD(T)/aug-cc-pVnZ (DK-CCSD(T)/aug-cc-pV*n*Z (*n*=D,T,Q) methods. Transition vectors corresponding to the KS DFT **TS** and CCSD(T) **TS** structures are shown. Distances are in Å, and angles are in degrees.

3. Tables S1-S3

Table S1. Relative energies (kJ/mol) of the ground $^1S(3d^{10}4s^2)$ state and the excited $^3P(3d^{10}4s^14p^1)$ and $^1P(3d^{10}4s^14p^1)$ states of the Zn atom calculated using single-reference[a] and multireference methods.

Method	$^1S(3d^{10}4s^2)$	$^3P(3d^{10}4s^14p^1)$	$^1P(3d^{10}4s^14p^1)$
non-relativistic			
MCSCF(2,4)[b]/aug-cc-pVTZ	0.0	337.6	544.8
MCQDPT2(2,4)[b]/aug-cc-pVTZ	0.0	366.1	560.2
CASPT2(2,4)[b]/aug-cc-pVTZ	0.0	364.0	552.7
CCSD(T)/aug-cc-pVTZ	0.0	369.9	
CCSD(T)/aug-cc-pVQZ	0.0	371.1	
CCSD(T)/aug-cc-pV5Z	0.0	371.5	
with scalar relativistic effects via second-order DK			
DK-MCSCF(2,4)[b]/aug-cc-pVTZ-DK	0.0	352.3	557.3
DK-MCQDPT2(2,4)[b]/aug-cc-pVTZ-DK	0.0	384.5	578.2
DK-CASPT2(2,4)[b]/aug-cc-pVTZ-DK	0.0	382.0	569.9
DK-CCSD(T)/aug-cc-pV5Z-DK	0.0	389.9	
Exp.[c]	0.0	389.5	559.4

[a]For Zn(^{3}P), this implies RCCSD(T).
[b]Active space used in the MCSCF, MCQDPT2 and CASPT2 calculations was 2 electrons in 4 orbitals, (2,4), arising from Zn 4s4p orbitals.
[c]Derived from Moore's tables (Ref.12 in the main text).

Table S2. Relative energies[a] of stationary points of the reaction of ground state atomic zinc with methane calculated at the ZPE-corrected CCSD(T)/aug-cc-pV*n*Z (*n*=T,Q,5) and DK-CCSD(T)/aug-cc-pV5Z-DK levels (in kJ/mol).

Species[c]	aug-cc-pVTZ[b]	aug-cc-pVQZ[c]	aug-cc-pV5Z[c]	aug-cc-pV5Z-DK[c,d]
Zn(^{1}S) + CH_4 (**1**)	0.0	0.0	0.0	0.0
Zn**…**H_3CH (**2**)	-1.6	-1.5	-1.5	-1.5
Zn**…**HCH_3 (**2a**)[e]	-1.4	-1.3	-1.2	-1.2
**TS**	333.0	334.3	335.1	341.0
$HZnCH_3$ (X^1A_1) (**3**)	46.9	45.2	45.2	47.7

[a]Relative to Zn(^{1}S) + CH_4 (**1**).
[b]At the geometries optimized at the CCSD(T)/aug-cc-pVTZ level and including the CCSD(T)/aug-cc-pVTZ vibrational zero-point energy (ZPE) contribution.
[c]At the geometries optimized at the CCSD(T)/aug-cc-pVQZ level and including the CCSD(T)/aug-cc-pVTZ ZPE contribution.
[d]Computed with the DK-CCSD(T) method.
[e]The geometry of van der Waals complex **2a** optimized at the CCSD(T)/aug-cc-pV*n*Z (*n*=D,T,Q) levels is shown in Figure S1.

Table S3. Relative energies[a] of stationary points of the reaction of excited ^3P state atomic zinc with methane calculated at the ZPE-corrected (R)CCSD(T)/aug-cc-pV*n*Z (*n*=T,Q,5) and DK-(R)CCSD(T)/aug-cc-pV5Z-DK levels[b] (in kJ/mol).

Species[c]	aug-cc-pVTZ[d]	aug-cc-pVQZ[e]	aug-cc-pV5Z[e]	aug-cc-pV5Z-DK[e,f]
$Zn(^3P) + CH_4$ (**1**)	369.9 (*0.0*)	371.1 (*0.0*)	371.5 (*0.0*)	389.9 (*0.0*)
$\mathbf{2^T}(^3A')$	360.7 (*-9.3*)	361.5 (*-9.4*)	362.3 (*-9.4*)	379.9 (*-10.0*)
$\mathbf{TS^T}(^3A')$	397.1 (*27.2*)	397.1 (*25.9*)	397.5 (*25.9*)	409.2 (*19.2*)
$HZnCH_3(^3A')$ ($\mathbf{3^T}$)	338.5 (*-31.4*)	339.3 (*-31.8*)	340.2 (*-31.4*)	346.0 (*-43.5*)
$\mathbf{TS_{diss}^T}(^3A')$	369.0 (*-1.3*)	369.0 (*-2.1*)	369.4 (*-2.1*)	374.9 (*-14.6*)
$ZnH(^2\Sigma^+) + CH_3(^2A_2'')$	341.4 (*-28.5*)	342.7 (*-28.5*)	343.5 (*-28.0*)	349.4 (*-40.6*)
$ZnCH_3(^2A_1) + H(^2S)$	366.5 (*-3.8*)	366.5 (*-4.6*)	366.9 (*-4.6*)	372.8 (*-17.2*)

[a]Relative to $Zn(^1S) + CH_4$ (**1**), except for the energies indicated in parentheses in italics which are relative to $Zn(^3P) + CH_4$ (**1**).
[b]For the open-shell species, this refers to the corresponding RCCSD(T) levels.
[c]For the geometries of the $ZnCH_3(^2A_1)$, $ZnH(^2\Sigma^+)$, and $CH_3(^2A_2'')$ radicals optimized at the RCCSD(T)/aug-cc-pV*n*Z (*n*=D,T,Q) levels, see Figure S5.
[d]At the geometries optimized at the CCSD(T)/aug-cc-pVTZ level and including the CCSD(T)/aug-cc-pVTZ vibrational zero-point energy (ZPE) contribution.
[e]At the geometries optimized at the CCSD(T)/aug-cc-pVQZ level and including the CCSD(T)/aug-cc-pVTZ ZPE contribution.
[f]Computed with the DK-CCSD(T) method.

4. Additional Computational Details

Optimization of molecular geometries and evaluation of harmonic vibrational frequencies at the (R)CCSD(T) level utilized numerical gradients and hessians. The Quadratic Steepest Descent (QSD) method was employed for locating minima and transition states at the (R) CCSD(T) level using the MOLPRO code (Ref.32 in the main text). For the (R)CCSD(T) geometry optimizations numerical gradients were computed with the step size of 0.01 bohr for distances and 1 degree for angles. The corresponding numerical Hessians were computed using the *central energy differences* with the step size of 0.01 a.u.
